# Analysis of orthopedic surgery of bone metastases in breast cancer patients

**DOI:** 10.1186/1471-2474-13-232

**Published:** 2012-11-27

**Authors:** Bernd Wegener, Marcus Schlemmer, Joachim Stemmler, Volkmar Jansson, Hans Roland Dürr, Matthias F Pietschmann

**Affiliations:** 1 Orthopedic Oncology, Department of Orthopedic Surgery, Ludwig-Maximilians-University Munich, Marchioninistrasse 15, D-81377, München, Gemany; 2 Medical Clinic III, Ludwig-Maximilians-University Munich, Marchioninistraße 15, 81377, Munich, Germany

**Keywords:** Breast cancer, Bone metastases, Surgical treatment of bone metastases

## Abstract

**Background:**

Breast cancer is the most common malignancy and the second leading cause of death in women. Because bone metastases are a common finding in patients with breast cancer, they are of major clinical concern.

**Methods:**

In 115 consecutive patients with bone metastases secondary to breast cancer, 132 surgical procedures were performed. Medical records and imaging procedures were reviewed for age, treatment of the primary tumor, clinical symptoms, surgical treatment, complications, and survival.

**Results:**

The overall survival of patients with metastatic breast cancer was dependent on the site and the amount of the metastases. Age was not a prognostic factor for survival. If the result of the orthopaedic surgery was a wide resection (R0) survival was significantly better than in the R1 (marginal resection – tumor resection in sane tissue) or R2 (intralesional resection) situation. Concerning the orthopaedic procedures there was no survival difference.

**Conclusion:**

In conclusion a wide (R0) resection and the absence of pathological fracture and visceral metastases were predictive for longer survival in univariate analysis. Age and the type of orthopaedic surgery had no impact on survival in multivariate analysis. The resection margins lost significance. The standard of care for patients with metastatic breast cancer to the bone requires a multidisciplinary approach.

## Background

Breast cancer is the most common malignancy and the second leading cause of death in women. It was estimated that approx. 180.000 women were newly diagnosed with breast cancer in the USA in 2008 and about 40.000 died of that disease [1]. The lifetime risk of developing invasive breast cancer in the USA is 12.6% (one out of eight women [2]. In autopsy studies, metastases in the skeleton occur at least as frequently as those in the lung [3]. It is well recognized that there is a relatively indolent course of the disease in many patients with predominantly bone metastases [4], while other patients suffer from severe pain [5]. Because bone metastases are a common finding in patients with breast cancer, they are of major clinical concern. In a population based study Wedin et al. found that of the breast cancer patients who presented with symptomatic skeletal metastases 17% needed surgery [6]. Pathologic fractures represent severe complications in these patients, especially fractures of the spinal vertebrae with spinal cord compression. The mean survival time of patients with bone metastases varies between 24 and 34 months [7,8]. With new systemic therapeutics available the survival time will increase and orthopaedic surgeons will see more breast cancer patients with bone metastases.

Different therapeutic options are available for treating symptomatic bone metastases, such as analgesics and systemic chemotherapy for pain relief as well as radiotherapy and prophylactic stabilisation for long term prevention of fractures [9]. In case of a pathological fracture surgery is inevitable in most cases. Patient selection is an important criterion regarding survival benefits and quality of life after surgical intervention. To evaluate prognostic factors on survival, a consecutive series of 115 patients with breast cancer, who were surgically treated for bone metastases in our institution, were reviewed. Clinical behaviour, surgical procedures, and treatment results were analyzed.

## Methods

Between January 1980 and September 2005, in 115 consecutive patients (112 women, 3 men) with bone metastases secondary to breast cancer 132 surgical procedures were performed. Medical records and imaging procedures were reviewed for age, treatment of the primary tumor, clinical symptoms, surgical treatment, complications, and survival. Statistical analyses were performed using the Cox regression for multivariate analysis, Kaplan-Meier life table analyses, and log-rank test for univariate analysis. The study was approved by the ethical committee.

## Results

The mean age of the 115 patients at the time of operation was 57.3 years (range: 30.6–83.6 years). Systemic hormone treatment was administered to 18 patients (16%), chemotherapy to 41 patients (36%), and radiation therapy to 61 patients (53%); some patients received a combination of the three therapies.

Nearly all patients presented with pain (98%); 72 patients (63%) had a pathologic fracture, fourteen patients (12%) had neurological impairments due to spinal compression. The mean duration of symptoms was 4.5 months (median: 2.9 months; range: 0–56 months). In 13 (11%) patients, breast cancer was diagnosed as a result of symptoms caused by osseous metastases. At presentation 9 (8%) patients had a solitary osseous lesion, 57 (49.5%) patients had more than one lesion, and 49 (42.5%) patients had an additional visceral involvement. The time from diagnosis of breast cancer to bone metastases surgery ranged from 0 to 30 years (mean: 5.7 years median: 4.3 years). Eighteen percent of the patients needed surgical therapy for bone metastases in the first year, 58% in the first 5 years, and 89% in the first 10 years. Fourteen patients (12%) had an interval longer than 10 years, nine of these were the ones who showed solitary bone matastases and were treated with wide resections.

**Figure 1:**
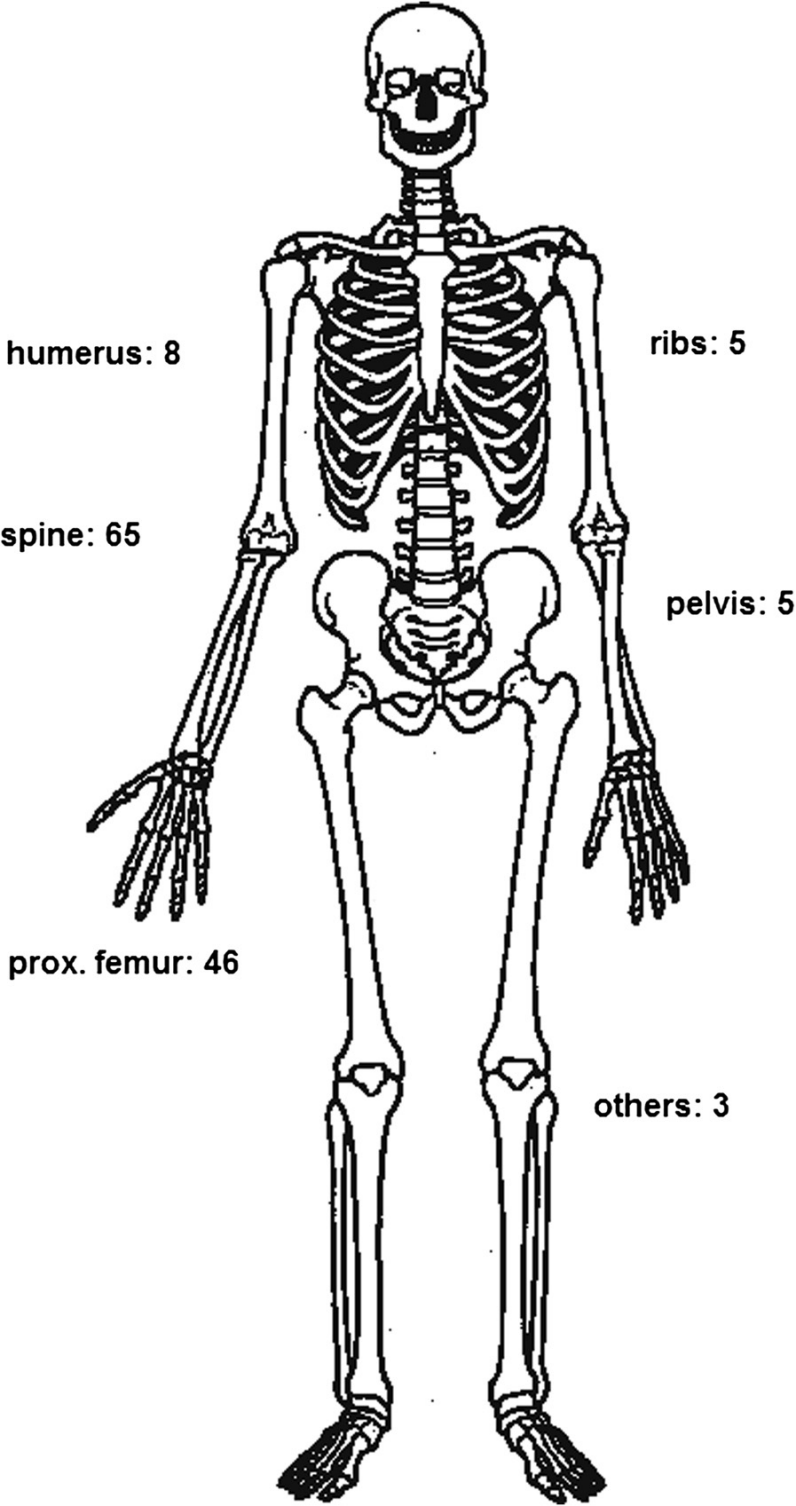
Location of 132 surgical procedures.

The locations of the surgical procedures are shown in Figure 1. The most common locations of bone metastases were the spine (65 patients) and the proximal femur (46 patients). (humerus: 8, pelvis: 5 and ribs: 5, others: 3)

Indications for treatment were untreatable pain, instability, impending fractures or fractures of the long bones and spinal compression because of tumor. Surgical therapy in the 115 patients varied depending on the site of the tumor, the extent of disease and the patient’s general health status.

Incisional biopsy was done in 14 (12%) patients. In one patient resection of the proximal humerus without reconstruction was done.

In 65 patients with involvement of the spine, 5 were treated with vertebroplasty and 4 with dorsal decompression only. In 32 patients dorsal instrumentation was used and in 17 patients a partial or complete vertebral resection with ventral stabilisation was done. In 7 patients only a CT-guided biopsy was taken for confirmation of diagnosis.

In 15 patients resection of the tumor and implantation of a tumor-endoprosthesis was performed, including one patient with an acetabular resection and reconstruction with a custom-made endoprosthesis. 19 patients received a standard hip arthroplasty, and one patient received a semiconstrained knee endoprosthesis. In 9 patients with solitary bone lesions without visceral tumor spread, a wide tumor resection was done. In 60 patients, as the intention was palliation, the surgical procedure was intralesional or marginal.

Fifteen patients (13%) suffered from complications associated with the surgical procedures. Among these complications deep venous thrombosis (n = 3), in one case with a severe embolic event, postoperative haematoma (n = 3) and failure of osteosynthesis (n = 3) were the most common. Furthermore we saw two wound healing complications, one non-union, one deep infection, one multi organ failure and in one case a persistent neurological deficit. Six patients died within the first 30 days after surgery. The median overall survival in our cohort was 17 months after diagnosis of bone metastases.

**Figure 2:**
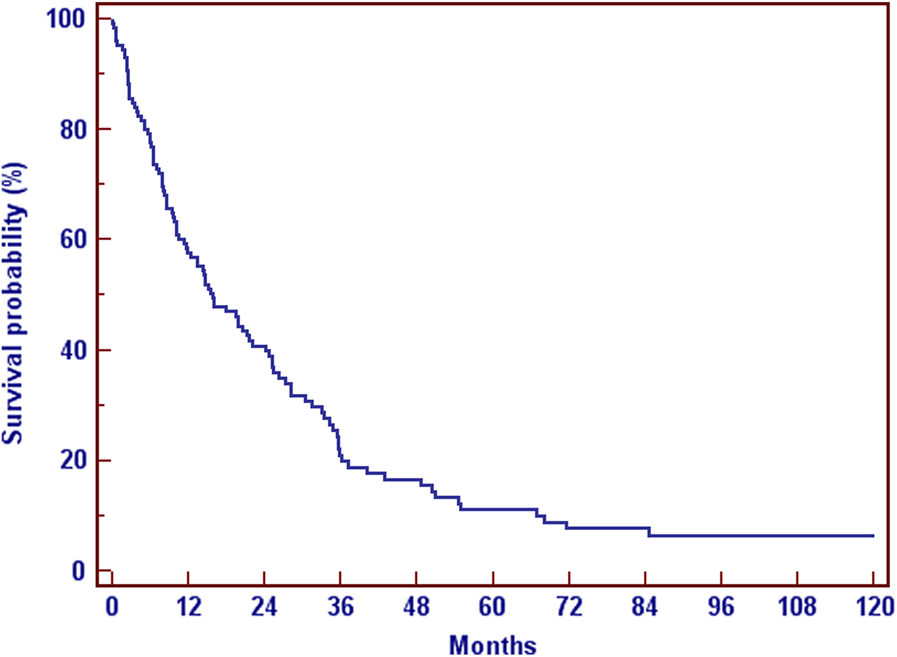
Overall survival.

**Figure 3:**
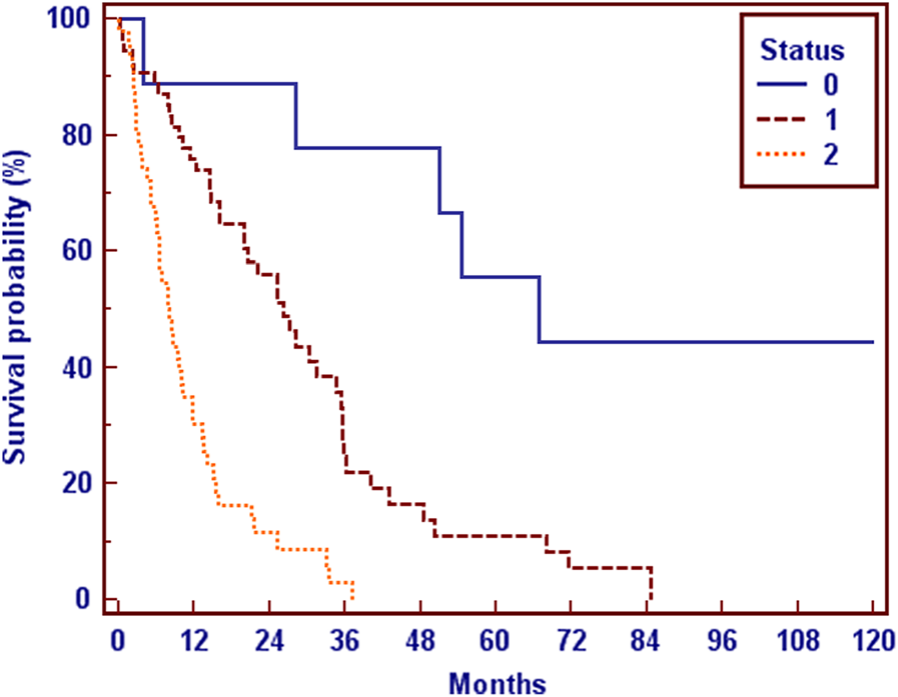
Survival and location of metastases: 0 = solitary bone metastase, 1 = multiple bone metastases, 2 = visceral metastases.

The overall survival of patients with metastatic breast cancer was dependent on the site and the amount of the metastases. Whereas patients with a solitary bone lesion had the best survival with a medium of 65 months, patients with visceral metastases had a medium survival of 13 months (Figure 2, Figure 3) after diagnosis of bone metastases.

**Figure 4:**
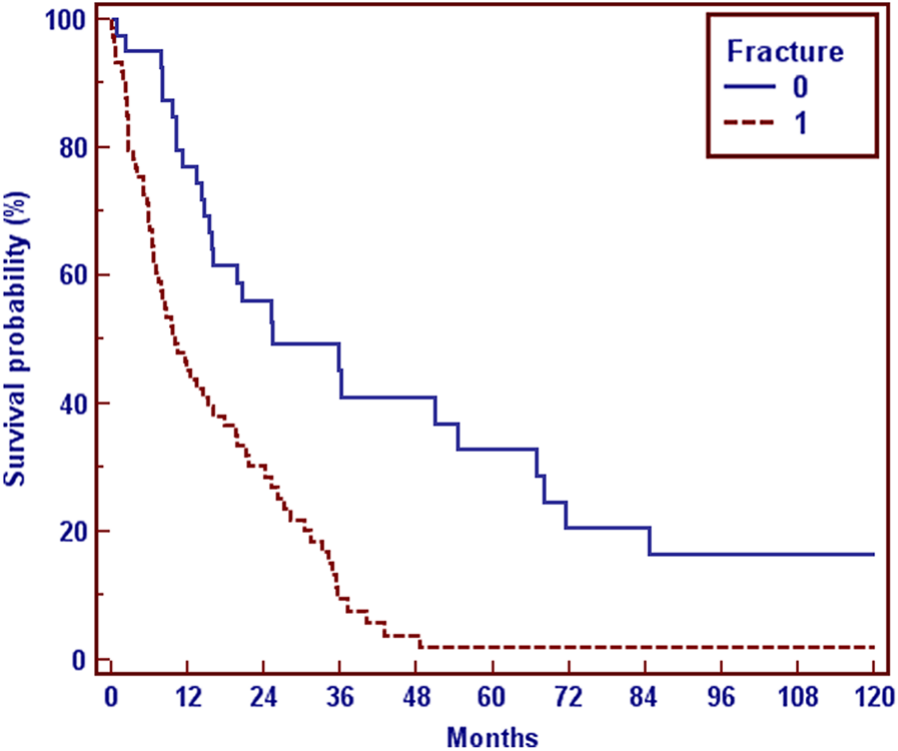
Survival and fracture, 1 = fracture, 0 = no fracture.

Age is not a prognostic factor of survival, as in the group of patients < 55 years or > 55 years no statistical difference could be shown. On the other hand patients with fracture of an extremity had an adverse median survival (10 months) in comparison to patients without fracture (25 months, Figure 4), which showed a statistical significance (p = 0.0001).

**Figure 5:**
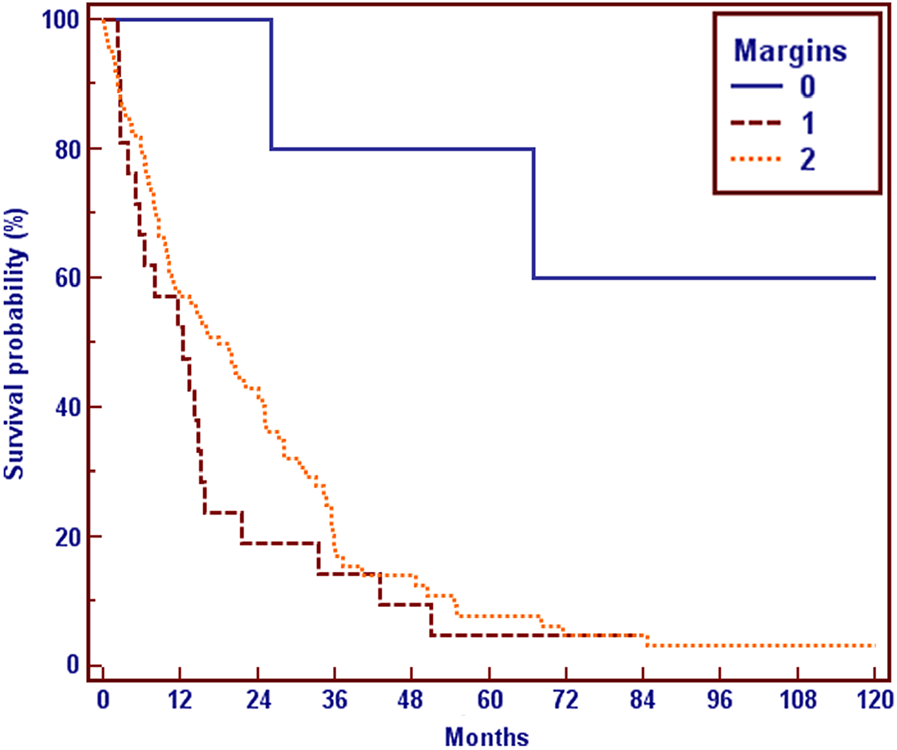
Survival and R-status 0 = R0, 1 = R1, 2 = R2.

If the result of the orthopaedic surgery was a wide resection (R0) survival was significantly (p = 0.0123) better than in the R1 or R2 situation (not reached vs 19 months vs 12 months, Figure 5).

Concerning the orthopaedic procedures there was no survival difference whether nail, SSTP (standard tumor prosthesis) or TTEP (tumor total endoprosthesis) was done (p = 0.1695).

In a multivariate approach to overall survival (Table 1) the margins and the fractures lost significance. Patients over 55 years showed now a slightly decreased prognosis, the dissemination of the disease proved to be the most important and highly significant influencing factor of overall survival.

**Table 1:** Multivariate analysis of overall survival in dependence to tumor dissemination (solitary bone, multiple bone, visceral), fracture (yes/no), Age (<=55 y/>55 y) and margins (R0, R1, R2)

**Covariate**	**SE**	**P**	**95%Cl of Exp(b)**
Tumor	0,2562	<0,0001	2,3939 to 6,5032
Dissemination			
Fracture	0,2576	0,5238	0,7131 to 1,9474
Age	0,01229	0,0116	1,0071 to 1,0565
Margin	0,2216	0,9564	0,6413 to 1,5219

## Discussion

We analyzed retrospectively 115 patients in the University Hospital of Munich, Germany who underwent 132 orthopedic surgery procedures because of metastatic breast cancer to evaluate prognostic factors of survival. Our cohort is heterogeneous as patients with limited disease or just one bone lesion and patients in the end stage of their disease with multiple lesions or fractures were analyzed. The time after first diagnosis of breast cancer until the diagnosis of bone metastases was median 4.3 years (0–30 years), which is in concordance with other cohorts [6].

Patients with metastatic breast cancer are in need of a systemic therapeutic approach as metastatic breast cancer is a systemic disease. Patients with absence of visceral metastases had an improved survival in contrast to patients with e.g. liver metastases. The combination of an antihormonal therapy, therapy with monoclonal antibodies in her-2 positive disease, cytotoxic agents and tyrosinkinase-inhibition may delay disease progression in these patients. Therefore the treatment of bone metastases, besides systemic treatment, includes different aspects as analgetics, bisphosphonates, radiation therapy and surgery [10]. Bisphosphonates are standard of care in prevention and treatment of osteolytic and osteoblastic bone metastases increasing the time to skeletal complications in patients with breast cancer [11-13]. Radiation therapy diminishes pain in most patients during ongoing treatment [14]. For patients without clinical benefit during radiation orthopedic surgery may be beneficial. An assessment of risk for pathological fractures should be implemented to perform prophylactic surgery [15]. Here a non-invasive CT (computed tomography)-based diagnosis can help oncologists and orthopaedic surgeons to predict fractures caused by breast cancer metastases [16].

The role of surgery in the palliative setting is to control pain or neurologic symptoms, stabilize fractures, improve function and remobilize patients. The location of the lesion or the complication guide surgical procedures. Large humeral or femoral segments can be reconstructed with endoprosthesis of new prosthetic materials [17,18]. We performed more endoprosthesis (27%) than osteosynthesis (17%), as these are beneficial for selected patients with metastatic tumors and bone loss, what we and others have shown before [19-22]. A wide resection (R0) could, on the first sight, significantly improve survival (p = 0.0123) in comparison to R1 or R2 resection. The median survival was not reached for the R0 group, in contrast to a median survival of 19 months in the R1 and 12 months for the R2 group. But those patients had a more limited disease and had been in a better general condition. They hence had been treated more aggressively. So we think this survival benefit is based on a selection bias. This is also proved by the multivariate analysis shown in Table 1.

Affection of the vertebra can be stabilized by “internal bracing” or screws in selected cases, allowing decompression of neural structures. As these procedures often do not lead to complete excision of the tumor lesion, delayed anterior approaches in combination with systemic therapy may be performed [23]. Attempts of local radical excision are meaningless in systemic metastatic breast cancer [24]. In an analysis of 125 surgical interventions in 87 breast cancer patients the surgical procedures to the spine provided pain relief and preservation or improvement of the neurological function [25]. Kyphoplasties showed to be a feasible option in 555 patients with 1150 vertebral fractures [26].

Pathologic fractures are strong indications for surgical procedures. The high percentage of patients with pathologic fractures (49%) in our series reflects, that our cohort had a poor prognosis. According to Bauer et al. we also found that a fracture of an extremity was a negative prognostic factor [27]. This could not be demonstrated for spinal fractures. The median survival for patients without fractures in comparison to patients with fractures was significantly longer (25 months vs. 10 months), which should encourage to perform orthopedic surgery in an earlier stage of disease to prevent fractures. The method of surgery (nail, SSTP or TTEP) had no influence on survival in our patients. The complication rate of 11% in our institution is low and in the range of other published series [25]. 9 patients with solitary bone metastases had been in the group of long survivors. We believe this is a bias. These patients had a more limited disease and had been in a better general condition and hence have been treated more aggressively.

A limitation of our study is caused by the long follow up period. Systemic and radiological therapies and protocols have changed over time. Therefore a differentiation of these parameters is not possible.

## Conclusions

In conclusion the possibility of a wide (R0) resection and the absence of pathological fractures and visceral metastases were predictive for longer survival, whereas age and the type of orthopaedic surgery had no impact on survival.

Due to new treatment modalities patients with metastatic breast cancer live longer, which alters the failure possibility of a surgical reconstruction. In these patients wide resection as performed in primary bone tumors, should be considered. This might encourage to use endoprosthetic rather than osteosynthetic techniques.

The standard of care for patients with metastatic breast cancer to the bone requires a multidisciplinary approach including radiotherapy, surgery and systemic treatment. Orthopedic surgeons should be active members in this multidisciplinary team with oncologists, radiologists and radiation-oncologists. Their part will become more important in the future as new operative techniques and materials will enable patients with metastatic breast cancer, who live longer because of better systemic approaches, to live with a better quality of life.

## Competing interests

The authors declare that they have no competing interests.

## Authors’ contributions

BW and MS have equally analyzed the data, created and edited the manuscript. JS and VJ as supervisors have checked the manuscript and helped in the creation of content. HRD and MFP managed the clinical care of patients and collected the statistical data retrospectively and revised the manuscript. All authors read and approved the final manuscript.

## Authors’ information

The Authors B. Wegener and M. Schlemmer contributed equally to prepare the manuscript.

## Pre-publication history

The pre-publication history for this paper can be accessed here:

http://www.biomedcentral.com/1471-2474/13/232/prepub
